# Cost Effectiveness of a Pharmacy-Only Refill Program in a Large Urban HIV/AIDS Clinic in Uganda

**DOI:** 10.1371/journal.pone.0018193

**Published:** 2011-03-28

**Authors:** Joseph B. Babigumira, Barbara Castelnuovo, Andy Stergachis, Agnes Kiragga, Petra Shaefer, Mohammed Lamorde, Andrew Kambugu, Alice Muwanga, Louis P. Garrison

**Affiliations:** 1 Pharmaceutical Outcomes Research and Policy Program, School of Pharmacy, University of Washington, Seattle, Washington, United States of America; 2 Infectious Diseases Institute, Makerere University, Kampala, Uganda; 3 Departments of Epidemiology and Global Health, School of Public Health, University of Washington, Seattle, Washington, United States of America; Erasmus University Rotterdam, Netherlands

## Abstract

**Background:**

HIV/AIDS clinics in Uganda and other low-income countries face increasing numbers of patients and workforce shortages. We performed a cost-effectiveness analysis comparing a Pharmacy-only Refill Program (PRP), a form of task-shifting, to the Standard of Care (SOC) at a large HIV/AIDS clinic in Uganda, the Infectious Diseases Institute (IDI). The PRP was started to reduce workforce shortages and optimize patient care by substituting pharmacy visits for SOC involving monthly physician visits for accessing antiretroviral medicines.

**Methodology/Principal Findings:**

We used a retrospective cohort analysis to compare the effectiveness of the PRP compared to SOC. Effectiveness was defined as Favorable Immune Response (FIR), measured as having a CD4 lymphocyte count of over 500 cells/µl at follow-up. We used multivariate logistic regression to assess the difference in FIR between patients in the PRP and SOC. We incorporated estimates of effectiveness into an incremental cost-effectiveness analysis performed from a limited societal perspective. We estimated costs from previous studies at IDI and conducted univariate and probabilistic sensitivity analyses. We identified 829 patients, 578 in the PRP and 251 in SOC. After 12.8 months (PRP) and 15.1 months (SOC) of follow-up, 18.9% of patients had a FIR, 18.6% in the PRP and 19.6% in SOC. There was a non-significant 9% decrease in the odds of having a FIR for PRP compared to SOC after adjusting for other variables (OR 0.93, 95% CI 0.55–1.58). The PRP was less costly than the SOC (US$ 520 vs. 655 annually, respectively). The incremental cost-effectiveness ratio comparing PRP to SOC was US$ 13,500 per FIR. PRP remained cost-effective at univariate and probabilistic sensitivity analysis.

**Conclusion/Significance:**

The PRP is more cost-effective than the standard of care. Similar task-shifting programs might help large HIV/AIDS clinics in Uganda and other low-income countries to cope with increasing numbers of patients seeking care.

## Introduction

The HIV/AIDS epidemic in Africa remains a global public health concern. New infections peaked in 1996 but the number of persons living with the disease, now 22.4 million, continues to rise, a result of a high rate of new infections and the life-saving and life-extending impact of antiretroviral therapy (ART) [1]. With the prevailing health workforce crisis [2], [3], HIV/AIDS clinics must find innovative ways to organize the way they provide care to numerous patients with a sub-optimal health workforce. The Infectious Diseases Institute (IDI), Makerere University, a regional center of HIV treatment, prevention, training, and research excellence in Kampala, Uganda, was faced with such a situation in 2006. Its out-patient clinic, which had 2,800 patients on ART in 2005, had grown to 10,000 total patients, half of whom were on ART and the number of patients was increasing without a substantial increase in clinical staff, particularly physicians. To alleviate the growing demand for physician visits and enable as many patients as possible to be initiated and maintained on therapy, IDI started a Pharmacy-only Refill Program (PRP).

The PRP was designed to substitute the prevailing Standard of Care (SOC) involving monthly physician visits with pharmacy-only monthly visits. Physicians selected patients for the PRP if they met the following criteria: 1) CD4 lymphocyte count greater than 200 cells/µL, 2) at least 12 months of ART, 3) self-reported adherence greater than 95%, 4) adherence to scheduled clinic visits for the preceding 6 months, 5) disclosed HIV status to spouse, 6) not pregnant, and 7) no substantial clinical event in the preceding 6 months. PRP-eligible patients picked up their antiretroviral medicines (ARVs) at the IDI pharmacy during monthly PRP visits without visiting a physician. However, PRP patients were asked screening questions by a pharmacy-based nurse during every PRP visit. It was arranged that PRP patients see a physician once every six months. Patients enrolled into the PRP and subsequently judged to have major clinical or social problems, or who developed problematic adherence to ART, were re-assigned to SOC. Therefore, the PRP did not replace SOC entirely.

The PRP is a form of task-shifting, the delegation of aspects of healthcare from more to less specialized health workers which has been proposed as a potential solution to the health workforce crisis in low-income countries [4], [5]. A systematic review of task-shifting in HIV/AIDS care concluded that it is an effective strategy for addressing shortages of health workers in Africa and that it offers high quality, cost-effective care to more patients than a physician-centered model [6]. Further evidence to support task-shifting has come from randomized trials which have found that nurse monitoring is non-inferior to doctor monitoring for the management of HIV patients in South Africa [7] and that task-shifting with persons living with AIDS, supported by personal digital assistants, results in similar health outcomes as the usual standard of care [8]. This is in addition to evidence from observational studies that suggests that task-shifting leads to improvements in access and good program outcomes for adults [9], [10] and children [11] and that that nurses can effectively and safely prescribe ART when given adequate training, mentoring, and support [12].

Yet despite the growing evidence of the potential role of task-shifting in improving HIV/AIDS treatment, policy action has been apathetic and some have argued that this is unethical [13]. Concerns about task-shifting have been raised by studies that show that the quality of care may suffer when non-physician clinicians perform physician duties [14] and a variety of other challenges remain, including addressing professional and institutional resistance, sustaining motivation and performance, and preventing deaths of health workers from HIV/AIDS [5].

Cost-effectiveness analysis considers both costs and health outcomes in evaluating the efficiency of healthcare interventions and allows policy makers to prioritize among competing uses of scarce healthcare resources. Cost-effectiveness studies might contribute to the policy dialogue surrounding HIV/AIDS care and improve the quality of policy decisions. However, we found only one study that assessed the costs of task-shifting [15] and none that assessed its potential cost-effectiveness. Therefore, we performed a study to estimate the cost effectiveness of the PRP – a form of task-shifting – as compared to SOC.

## Methods

### Study design

We performed a retrospective cohort analysis and an incremental cost-effectiveness analysis.

### Retrospective cohort study

Using data from the IDI clinic database we retrospectively identified a cohort of patients treated at IDI in 2005, 2006, and 2007. We defined the exposed (to the PRP) group as patients who were enrolled in the PRP program in the first 6 months of its initiation starting in June 2006 and the unexposed (SOC) group as patients that 1) had reached a CD4 lymphocyte count of 200 cells/µl after 1 year on ART and 2) after reaching a CD4 lymphocyte count of 200 cells/µl were followed for at least one year before the start of the PRP program. The SOC patients were selected from the pre-PRP cohort so as to obtain a group of patients with similar characteristics. We started following PRP patients from initiation into the program and SOC patients from the first visit after achieving a CD4 lymphocyte count of 200 cells/µl. We excluded patients who had been on ART for less than one year and patients who were lost to follow-up during the follow-up period. The main outcome of our evaluation was a binary variable—whether or not patients had a favorable immune response (FIR), measured as follow-up CD4 lymphocyte count over 500 cells/µl at follow-up. This cut-off point is the lower limit of normal for Ugandan populations [16].

Other outcomes included median increase in CD4 lymphocyte count at follow-up and proportion of patients in different CD4 lymphocyte count ranges. We assessed outcomes as recorded in the medical records at the clinic visit at which a CD4 lymphocyte count was available and that was closest in date to 1 year after initiation of ART. We recorded all available covariates from the clinic records, i.e., age, gender, duration of ART, initial ART regimen, current ART regimen, presence/absence of opportunistic infection or neoplasm at baseline and follow-up, self-reported adherence (visual analog scale), CD4 lymphocyte count at start of ART, and CD4 lymphocyte count at baseline.

### Statistical analysis

We used 2-tailed tests and an α-level of 0.05 for all our analyses. We present descriptive statistics as differences between patients in the PRP and SOC groups using the student t-test for means and chi-square test for proportions. Logistic regression was used to estimate the odds ratios (ORs) of having a FIR. The ORs are from unadjusted models (crude ORs) and from models adjusted for possible confounders regardless of statistical significance at univariate analysis. We performed statistical analyses using STATA 9.1, College Station TX.

### Determination of costs

We separated the cost of implementing the PRP and SOC into the following categories: ART, other drugs, radiology, laboratory tests, health personnel, overhead and capital, patient transport, and lost patient time. We obtained costs of ART, other drugs, laboratory tests and radiology from a previous study at IDI in which investigators conducted a retrospective review of medical records to estimate resource utilization [17]. We obtained costs of health personnel and lost patient time from previous studies at IDI which included a time-and-motion survey to estimate health worker and patient time use [18] and a cost-minimization study in which data on health worker and patient wages were combined with data from the time-and-motion survey [15]. We obtained overhead and annualized capital costs from the World Health Organization Choosing Interventions that are Cost-Effective (WHOCHOICE) database for Uganda [19]. The annual costs were as follows: ART, $243; other drugs, $35; radiology, $2; laboratory tests, $34; health personnel (PRP), $10; health personnel (SOC), $31; overhead and capital costs, $141; patient transport, $20; lost patient time (PRP), $4; and lost patient time (SOC), $16. All costs are in 2009 US dollars.

### Cost-effectiveness analysis

We performed an incremental cost-effectiveness analysis from the “limited” societal and Ministry of Health (MoH) perspectives. The limited societal perspective [20] refers to analyses which do not meet the full criteria of the reference case as defined by the Panel on Cost-Effectiveness [21]. In our study, this perspective included all the different cost categories described above (direct medical and direct non-medical costs) but did not include productivity losses due to morbidity and mortality (indirect costs). The MoH perspective was included because the MoH is the relevant payer in Uganda since the country has a national health service that, in theory, should provide health care to all citizens. The MoH perspective only included direct medical costs and excluded direct non-medical costs (patient transport and lost patient time). The outcome of the analysis was cost per FIR over the 13-month follow-up period. This intermediate outcome was used in the analysis because patients in our study population have not been followed over a long-enough time period to allow for a more appropriate outcome to be measured.

### Uncertainty analysis

To ascertain the robustness of our results, we performed one-way and probabilistic sensitivity analyses. Cost estimates were halved and doubled and probabilities were reduced or increased by 20%. We created probability distributions for all of the parameters (probability of FIR and costs) in the model. For different parameters we used the baseline value for the mean, and estimated the standard error based on the approximation that the range used for the one-way sensitivity analysis represented a 95% confidence interval. We used a beta distribution for proportions and a gamma distribution for costs. We used Monte Carlo simulation to create 10,000 samples for which expected values were calculated. We examined a scatter plot of incremental cost and effectiveness pairs on the cost-effectiveness plane to examine the relative proportion that lay in the different quadrants. The scatter plot was also used to examine the uncertainty surrounding whether or not the PRP would be cost-effective and at what value it would be cost-effective. To summarize this uncertainty and better estimate potential decision uncertainty, we also calculated the proportion of iterations for which the PRP was cost-effective relative to SOC varying limits of cost-effectiveness (willingness to pay), and generated a cost-effectiveness acceptability curve. Cost-effectiveness analysis was performed using TreeAge Pro, TreeAge Software Inc, Williamstown, MA.

The study was approved by the Makerere University Faculty of Medicine Research and Ethics Committee (number 2009-120) and the Uganda National Council for Science and Technology (number HS 683). The Ethics Committee has approved the performance of evaluations using secondary clinic data without patient consent and has set standards for maintaining confidentiality including analysis after stripping data of unique personal identifiers, holding charts in secure locked locations, and protecting databases with passwords accessible to study staff members only.

## Results

### Baseline characteristics

We enrolled 829 patients in the analysis, 578 in the PRP group and 251 in SOC group. Table 1 shows the descriptive demographic, clinical and laboratory characteristics of the cohorts by exposure status. PRP patients were followed for a significantly shorter period of time (12.8 vs. 15.1 months; p-value<0.001), were older (38.8 vs. 35.7 years; p-value<0.001), had been on ART longer (41.8 vs. 30.9 months; p-value<0.001) and had lower baseline CD4 lymphocyte counts (218 vs. 292 cells/µl; p-value<0.001). PRP and SOC patients were also significantly different by initial and current ART regimen, presence or absence of opportunistic infections at baseline and follow-up, and proportion with sub-optimal adherence. The exposure groups were not different by gender and CD4 lymphocyte count at start of ART.

**Table 1:** Demographic, clinical and laboratory characteristics of the study population by method of follow-up^a^.

Category	Sub-category	SOC (%)	PRP (%)	Total (%)	p-value
Time (baseline to follow-up)		12.8 (1.6)	15.1 (1.3)	13.5 (1.9)	<0.001
Age, years		38.8 (7.5)	35.9 (7.5)	36.8 (7.6)	<0.001
Gender	Male	100 (39.8)	253 (43.8)	353 (42.6)	0.293
	Female	151 (60.2)	325 (56.2)	476 (57.4)	
ART duration (months)		41.8 (16.2)	30.9 (13.0)	34.2 (14.9)	<0.001
Initial ART regimen	d4T-3TC-NVP	160 (63.8)	362 (62.6)	522 (63.0)	<0.001
	ZDV-3TC-EFV	51 (20.3)	194 (33.6)	245 (11.2)	
	Other*	40 (15.9)	22 (3.8)	62 (7.5)	
Current ART regimen	ZDV-3TC-NVP	52 (20.7)	167 (29.9)	219 (26.4)	<0.001
	ZDV-3TC-EFV	37 (14.7)	154 (26.6)	191 (23.0)	
	ZDV-TDF-FTC-LPV/r	33 (13.5)	165 (28.6)	198 (23.9)	
	Other**	129 (51.4)	92 (15.9)	221 (26.6)	
OI at baseline	None	216 (86.1)	544 (94.1)	760 (91.7)	<0.001
	1 or more	35 (13.9)	34 (5.9)	69 (8.3)	
OI at follow-up	None	220 (93.4)	540 (87.6)	760 (91.7)	0.006
	1 or more	31 (6.6)	38 (12.4)	69 (8.3)	
AdherenceΨ	<95%	26 (11.1)	9 (1.6)	35 (4.3)	<0.001
	>95%	208 (88.9)	564 (98.4)	772 (95.7)	
CD4 count (start of ART)		121 (131)	124 (103)	123 (112)	0.758
CD4 count (start of study)		218 (160)	292 (145)	268 (154)	<0.001

a All data are n (%) or mean (SD).

*Includes d4T-3TC-EFV, ZDV-3TC-NVP, NVP-TDF-3TC, ZDV-ddI-LPV/r, ZDV-3TC-LPV/r, ddI-d4T-LPV/r, ZDV-EFV-LPV/r, 3^rd^ line drugs and unknown drugs.

**Includes D4T-3TC-NVP, D4T-3TC-EFV, TDF-FTC-EFV, NVP-TDF-3TC, TDF-FTC-NVP, ZDV-DDI-LPV/r, ZDV-3TC-LPV/r, DDI-D4T-LPV/r, TDF-FTC-LPV/r, ZDV-TDF-LPV/r, ZDV-3TC-DDI-LPV/r , TDF-EFV-LPV/r, 3TC-NVP-LPV/r and other 3^rd^ line drugs.

Ψ N = 807.

SOC: standard of care; PRP: Pharmacy Refill Program; ART: antiretroviral treatment; d4T: stavudine; 3TC: lamivudine; NVP: nevirapine; ZDV: zidovudine; EFV: efavirenz; TDF: tenofovir; FTC: emtricitabine; LPV/r: lopinavir/ritonavir; OI: opportunistic infection.

### Immune response

At baseline, 8.1% of cohort members had a CD4 lymphocyte count above 500 cells/µl, 5.7% in the PRP group and 9.5% in the SOC group. At follow-up, 18.9% of cohort members had a FIR, 19.6% in the PRP group and 18.6% in the SOC group. Median CD4 lymphocyte count increase between baseline and follow-up was 53 cells/µl in the PRP group and 128 cells/µl in the SOC group. At follow-up the proportion of patients with CD4 less than 200 cell/µl, 200–350 cells/µl, 350–500 cells/µl and above 500 cells/µl were 30.0%, 15.1%, 39.4%, 25.5%, 19.9% respectively in the PRP group and 14.6%, 40.2%, 26.7%, 18.6% respectively in the SOC group. Table 2 shows the results of the univariate and multivariate logistic regression analysis comparing PRP and SOC. There was a non-significant 9% decrease in the odds of having a FIR for PRP compared to SOC after adjusting for other variables.

**Table 2:** Univaraite and multivariate logistic regression analysis of variables associated with follow-up CD4 cell count over 500 cells/ul (n = 807).

Variable	Sub-category	Unadjusted OR (95% CI)	p-value	Adjusted OR (95% CI)	p-value
Exposure status	SOC	1 [Reference]		1 [Reference]	
	PRP	0.93 (0.72–1.60)	0.737	0.93 (0.55–1.58)	0.797
Duration of follow-up	<1 year	1 [Reference]		1 [Reference]	
	>1 years	1.53 (1.01–2.33)	0.045	1.98 (1.19–3.25)	0.007
Duration of ART	<2 years	1 [Reference]		1 [Reference]	
	2–3 years	1.12 (0.66–1.90)	0.682	0.84 (0.47–1.52)	0.570
	>3 years	0.56 (0.33–0.96)	0.035	0.34 (0.18–0.65)	<0.001
Age		1.02 (0.99–1.05)	0.072	1.02 (0.99–1.04)	0.286
Gender	Male	1 [Reference]		1 [Reference]	
	Female	0.44 (0.29–0.66)	<0.001	0.47 (0.30–0.73)	<0.001
Initial ART regimen	d4T-3TC-NVP	1 [Reference]		1 [Reference]	
	ZDV-3TC-EFV	1.61 (1.03–2.52)	0.035	2.45 (0.81–7.35)	0.109
	Other*	1.01 (0.49–2.00)	0.988	1.09 (0.47–2.54)	0.833
Current ART regimen	ZDV-3TC-NVP	1 [Reference]		1 [Reference]	
	ZDV-3TC-EFV	1.48 (0.86–2.52)	0.152	0.62 (0.18–2.11)	0.442
	ZDV-TDF-FTC-LPV/r	0.99 (0.61–1.64)	0.952	1.03 (0.61–1.85)	0.903
	Other**	1.51 (0.89–2.53)	0.120	1.68 (0.91–3.11)	0.098
OI at baseline	None	1 [Reference]		1 [Reference]	
	1 or more	1.62 (0.76–3.49)	0.214	1.68 (0.75–3.79)	0.210
OI at follow-up	None	1 [Reference]		1 [Reference]	
	1 or more	0.85 (0.44–1.61)	0.611	0.83 (0.40–1.69)	0.602
Adherence ^Ψ^	Sub-optimal	1 [Reference]			
	Optimal	1.33 (0.59–3.01)	0.490	1.37 (0.55–3.39)	0.501
CD4 count at start of ART	<200	1 [Reference]		1 [Reference]	
	200–300	0.44 (0.28–0.69)	0.001	0.44 (0.27–0.72)	<0.001
	>300	0.36 (0.19–0.69)	0.002	0.39 (0.19–0.78)	0.008

OR: odd ratio; SOC: standard of care; PRP: Pharmacy Refill Program; ART: antiretroviral treatment; d4T: stavudine; 3TC: lamivudine; NVP: nevirapine; ZDV: zidovudine; EFV: efavirenz; TDF: tenofovir; FTC: emtricitabine; LPV/r: lopinavir/ritonavir; OI: opportunistic infection.

### Cost effectiveness analysis

Table 3 shows the results of the cost-effectiveness analysis. The mean costs were lower in the PRP group than in the SOC group ($520 vs. $655 from the societal perspective and $496 vs. $610 from the MoH perspective). The probability of FIR was also lower in the PRP group compared to the SOC group (0.186 vs. 0.196). The incremental cost-effectiveness ratio for PRP compared to SOC was $13,500 per FIR from the societal perspective and $11,400 from the MoH perspective. These ICERs lie in the “southwest” quadrant of the cost-effectiveness plane and may be interpreted as follows: the PRP leads to one less FIR than the SOC at an incremental savings of $13,500 from the limited societal perspective or $11,400 from the MoH perspective.

**Table 3:** Mean and incremental costs, probability of CD4 cell count over 500 cell/ul at 1 year and cost-effectiveness comparing PRP and standard care in patients on antiretroviral treatment at the IDI clinic, Kampala, Uganda.

	SocietalCost*(US$)	Inc.	HealthcareCost*	Inc.	Probability of FIR	Inc.	Limited Societal ICER (US$/FIR)	MoH ICER (US$/FIR)
SOC	655	–	610	–	0.196	–	–	
PRP	520	−135	496	−114	0.186	−0.010	13,500	11,400

Inc. – Incremental; ICER: Incremental Cost-Effectiveness Ration; FIR – Favorable Immune Response; PRP: Pharmacy-Only Refill Program; SOC – Standard of Care.

*All costs are per person per year.

**Figure 1:**
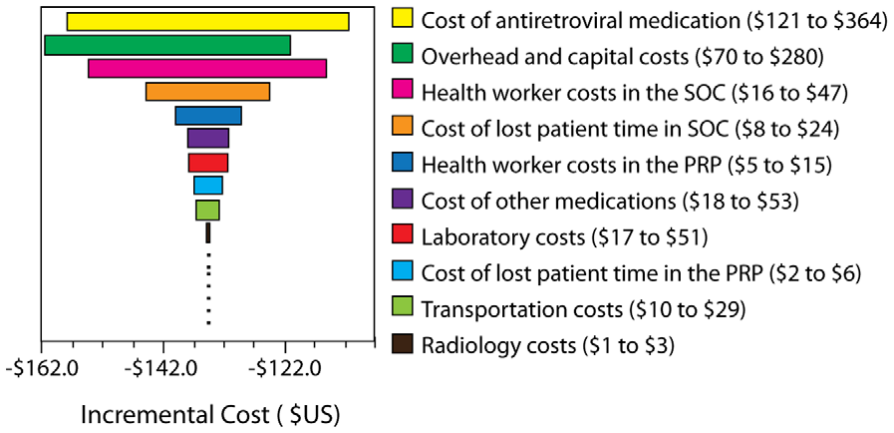
Tornado diagram of univariate sensitivity analysis showing the impact on incremental costs comparing Pharmacy-only Refill Program (PRP) versus Standard of Care (SOC).

Univariate sensitivity analyses showed that the incremental cost (Figure 1) was most sensitive to the cost of antiretroviral medication and that the incremental effectiveness was most sensitive to probability of favorable immune response.

**Figure 2:**
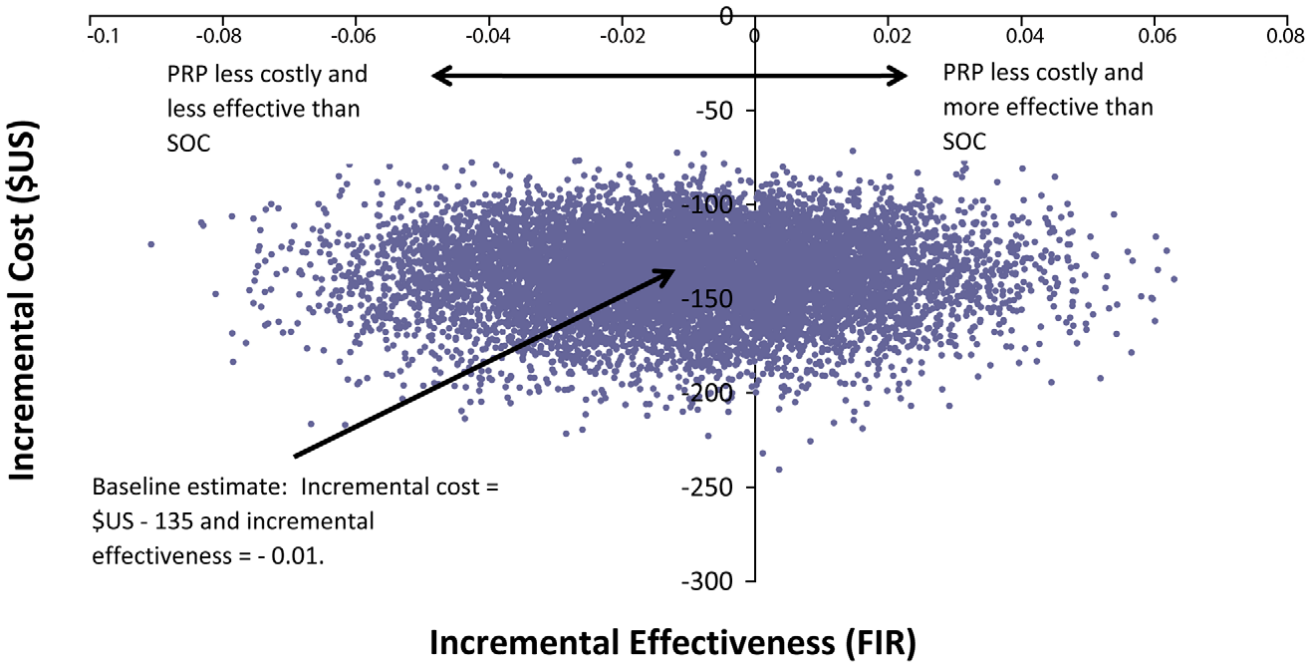
Scatter plot of estimated joint density of incremental costs and incremental effects of Pharmacy-only Refill Program (PRP) versus Standard of Care (SOC) by Monte Carlo simulation.

Figure 2 is a scatter plot that illustrates the uncertainty in the expected incremental cost and FIR. All Monte Carlo replicates comparing PRP to SOC lie below zero on the cost axis, indicating a high degree of certainty that PRP is less costly than SOC. Data points that lie in the “southwest” quadrant of the cost-effectiveness plane represent a loss in the probability of FIR at a decreased cost for PRP compared to SOC. Points that lie in the “southeast” quadrant represent a gain in the probability of FIR at a decreased cost of PRP compared to SOC. The spread of points in the vertical axis indicates some uncertainty in the magnitude of cost savings attributed to PRP.

With regard to effectiveness, the location and spread of the points indicate a high degree of uncertainty in the existence and extent of the reduction in benefit in FIR comparing PRP to SOC at follow-up. This is consistent with the finding of a non-significant decrease in odds of FIR for PRP compared to SOC in the multivariate logistic regression analysis.

**Figure 3:**
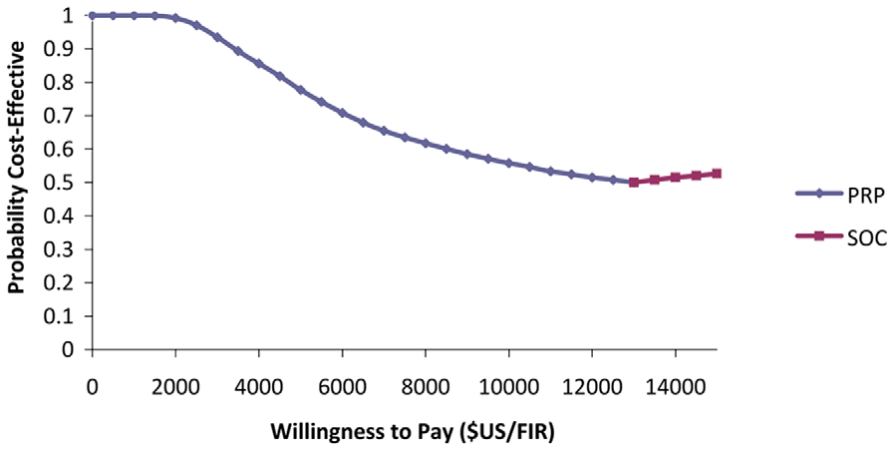
Cost-effectiveness acceptability curve showing the probability that Pharmacy-only Refill Program (PRP) or Standard of Care (SOC) is cost-effective compared to the other over a range of values of willingness to pay.

Figure 3 shows the results of the probabilistic sensitivity analysis presented as a cost-effectiveness acceptability curve. It indicates that, at low levels of willingness to pay, PRP is cost-effective in a larger proportion of iterations compared to SOC. The situation changes in favor of SOC at a willingness to pay of approximately $13,000 per FIR.

## Discussion

Using a retrospective cohort study and incremental cost-effectiveness analysis, we performed an evaluation of the Pharmacy-only Refill Program (PRP) in a large urban HIV clinic in Kampala, Uganda. Our study suggests that, judging from FIR measured as the proportion of patients who have a CD4 lymphocyte count over 500 cells/µl at follow-up, the PRP was not significantly different from SOC and was more cost-effective. The results were robust to univariate and probabilistic sensitivity analysis. Our findings represent a common situation in low-income countries—a healthcare policy intervention that results in a slight loss in effectiveness but leads to cost savings. We found that the PRP would lead to one less FIR than the SOC at an incremental savings of $13,500 from the limited societal perspective and $11,400 from the MoH perspective. This is a substantial amount of money in a country with a per capita expenditure on health care of less than US$30 [22]. With such a severe budget constraint, the rational choice may be to implement the PRP, particularly given the evidence of a non-significant reduction in FIR at multivariate analysis.

A key strength of our study was the combination of an impact evaluation based on a retrospective cohort study with a cost-effectiveness analysis supported by probabilistic sensitivity analysis. The impact evaluation showed that there was a small and not statistically significant increase in odds of FIR at follow-up for the SOC. Based on effectiveness alone, one might conclude that either follow-up strategy is equally effective. But after performing the cost-effectiveness analysis and sensitivity analysis, PRP appears to be the better strategy because of the lower cost compared to SOC despite the lack of a statistically significant difference in FIR between the two strategies. We also quantified decision uncertainty around the estimate of incremental cost-effectiveness using a cost-effectiveness acceptability curve. This is particularly important give the results of the impact evaluation showing no difference between PRP and SOC.

Our study had weaknesses that we propose as caveats to the interpretation of our results. We used an intermediate outcome—CD4 lymphocyte count. While this outcome is a reasonable measure of clinical progress in HIV/AIDS patients, the optimal study would follow patients over their lifetime and compare life-years. In addition to lifetime follow-up, the ideal study would also assess the patients' satisfaction and quality of life which were beyond the scope of our study.

Additionally, despite performing a multivariate analysis, the more favorable outcomes of our PRP patients may well reflect residual selection bias. Only patients who fulfilled all of the criteria were enrolled into the PRP. Some of their unmeasured characteristics may affect our effectiveness estimate. A more formal assessment of the relative effectiveness of PRP in ART management would require a randomized controlled trial. A recent randomized controlled trial in South Africa found that nurses were non-inferior to doctors when monitoring the treatment of HIV patients on ART [7]. Another cluster randomized trial in Uganda found that patients receiving home-based support, monitoring, and drug delivery by lay workers with 6-monthly routine evaluation achieved favorable and comparable outcomes to patients receiving facility-based care with monthly visits for drug refill and 3-monthly evaluation [23]. We found no trial directly comparing doctor follow-up to pharmacy-only follow-up.

Other studies have found that non-physician care, also called task-shifting, in low-income countries achieves favorable and comparable outcomes to physician care [7], [8], [9], [10], [11], [12] and may achieve cost savings [15]. To our knowledge, this is the first cost-effectiveness evaluation of a pharmacy refill program in this setting and the first evaluation of non-physician care to include costs and outcomes in the same analysis.

In conclusion, our study suggests that a pharmacy-only refill program may be a viable and efficient service delivery option for delivery of ART to eligible patients in Uganda and other low-income countries which are seeking innovative ways to optimize resource allocation to large patient populations, particularly in the face of the current crisis of health workers. Practitioners and clinic managers, as well as policy makers in this setting might consider similar programs or start discussions to widely implement them.
